# Azoxymethane Alters the Plasma Metabolome to a Greater Extent in Mice Fed a High-Fat Diet Compared to an AIN-93 Diet

**DOI:** 10.3390/metabo11070448

**Published:** 2021-07-09

**Authors:** Huawei Zeng, Shahid Umar, Zhenhua Liu, Michael R. Bukowski

**Affiliations:** 1 Grand Forks Human Nutrition Research Center, Agricultural Research Service, United States Department of Agriculture, Grand Forks, ND 58203, USA; michael.bukowski@usda.gov; 2 Department of Surgery and University of Kansas Cancer Center, Kansas City, KS 66160, USA; sumar@kumc.edu; 3 School of Public Health and Health Sciences, University of Massachusetts, Amherst, MA 01003, USA; zliu@nutrition.umass.edu

**Keywords:** aberrant crypt foci, azoxymethane, colon cancer, high-fat diet, inflammation, metabolome, obesity

## Abstract

Consumption of a high-fat diet (HFD) links obesity to colon cancer in humans. Our data show that a HFD (45% energy fat versus 16% energy fat in an AIN-93 diet (AIN)) promotes azoxymethane (AOM)-induced colonic aberrant crypt foci (ACF) formation in a mouse cancer model. However, the underlying metabolic basis remains to be determined. In the present study, we hypothesize that AOM treatment results in different plasma metabolomic responses in diet-induced obese mice. An untargeted metabolomic analysis was performed on the plasma samples by gas chromatography time-of-flight mass spectrometry (GC-TOF-MS). We found that 53 of 144 identified metabolites were different between the 4 groups of mice (AIN, AIN + AOM, HFD, HFD + AOM), and sparse partial least-squares discriminant analysis showed a separation between the HFD and HFD + AOM groups but not the AIN and AIN + AOM groups. Moreover, the concentrations of dihydrocholesterol and cholesterol were inversely associated with AOM-induced colonic ACF formation. Functional pathway analyses indicated that diets and AOM-induced colonic ACF modulated five metabolic pathways. Collectively, in addition to differential plasma metabolomic responses, AOM treatment decreases dihydrocholesterol and cholesterol levels and alters the composition of plasma metabolome to a greater extent in mice fed a HFD compared to the AIN.

## 1. Introduction

Consumption of a high-fat diet (HFD) links obesity to colon cancer in humans [1,2]. The global obesity epidemic has, in part, been attributed to the adoption of Western lifestyle practices, including increased consumption of high-energy diets [1,2,3]. Diet-induced obesity is now established as a risk factor for cancer, and overweight and obesity affect two-thirds of Americans and an estimated 2.3 billion people worldwide [4]. In mice, consumption of a HFD can lead to the accumulation of excess body fat that is associated with adipose tissue dysfunction and a chronic state of low-grade inflammation known to promote tumor development [5,6]. However, there are few mechanistic studies on early colonic tumorigenesis concerning the underlying metabolic regulation in the context of HFD-induced obesity.

We recently reported that in a mouse model, a HFD promotes colonic aberrant crypt foci (ACF, putative preneoplastic lesions) formation accompanied by increased systemic levels of proinflammatory cytokines [7]. We therefore posit that determining plasma metabolomic responses may lead to a greater understanding of the metabolic regulation concerning a HFD and colonic ACF formation. As small-molecule metabolites exhibit an organisms’ physiological condition at a given moment, these metabolites represent a dynamic situation in response to biochemical and pathological changes [8,9]. Thus, untargeted metabolite profiling (e.g., metabolomics) is an effective approach that aids in determining these underlying biochemical changes [10]. It is known that a HFD promotes AOM-induced ACF formation in a mouse model, and differential metabolites were identified in the serum of colon cancer patients compared to the healthy controls [7,11,12]. Therefore, we hypothesize that AOM treatment may cause different plasma metabolomic responses in AOM mouse colon cancer models fed with the HFD compared to the AIN.

## 2. Results

### 2.1. Identified Metabolites and Their Group Separation

The plasma samples were collected at the end of the study (Week 14) [7]. We identified 144 metabolites (Table S1) from 555 discrete signals detected in the plasma by using GC-TOFMS. There were 53 of 144 metabolites significantly different between the 4 groups of mice (AIN, AIN + AOM; HFD, HFD + AOM), and the relative values for these 53 metabolites compared to the AIN group are shown in Table 1.

**Figure 1:**
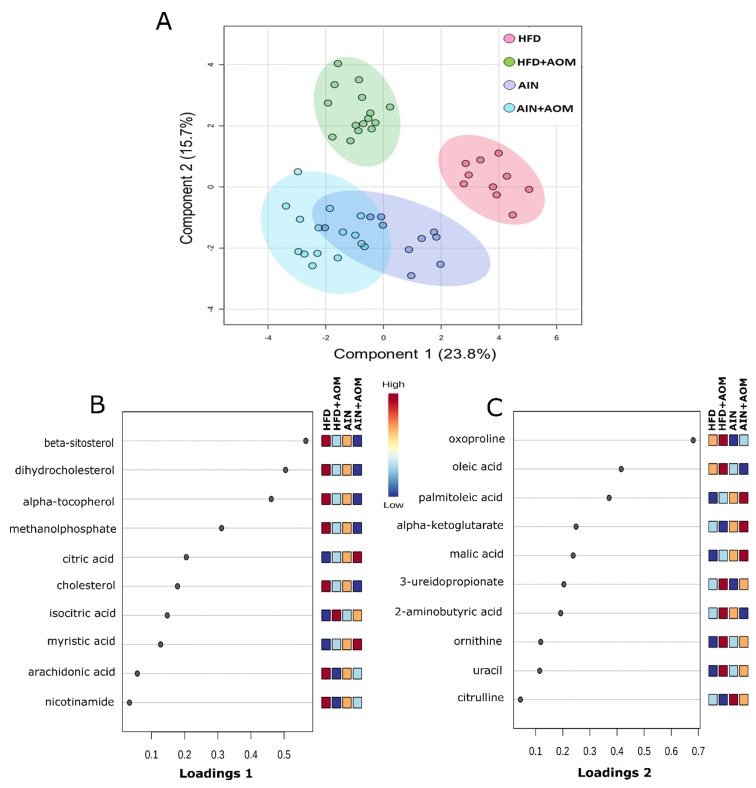
Two-dimensional (2D) sPLS-DA of the 4 experimental groups (**A**) and loading plots of 10 metabolites that are most significant in group separation among the four groups for Component 1 (**B**) and Component 2 (**C**). Mice without AOM treatment (control), *n* = 10/group (AIN or HFD group). Mice with AOM treatment (AIN + AOM or HFD + AOM group), *n* = 15/group.

To examine the diet-AOM effect on metabolome, sparse partial least-squares discriminant analysis (sPLS-DA) was used to visualize the metabolite group separation and individual sample variability (Figure 1A). We found that there was a separation between the HFD and HFD + AOM groups but not the AIN and AIN + AOM groups.

The 10 major metabolites influencing separation along Component 1 were (1) beta-sitosterol (>120% increase), dihydrocholesterol (>89% increase), alpha-tocopherol (>94% increase), methanol phosphate (>18% increase), cholesterol (>32% increase), arachidonic acid (>6% increase), and nicotinamide (>58% increase), which were the highest concentrations in the HFD group compared to the other three groups; (2) the concentrations of citric acid, isocitric acid and myristic acid were decreased by (>23%) in the HFD group compared to the other three groups (Figure 1B).

The 10 major metabolites influencing separation along Component 2 were: (1) oxoproline (>29% increase), oleic acid (>72% increase), 3-ureidopropionate (>67% increase), 2-aminobutyric acid (>58% increase), ornithine (>65% increase), and uracil (>25% increase) were the highest concentration in the HFD + AOM group compared to the otherthree3 groups; (2) palmitoleic acid (>30% decrease), alpha-ketoglutarate (>28% decrease), malic acid (>40% decrease), and citrulline (>30% decrease) exhibited a lower concentration in the HFD and HFD + AOM groups compared with the AIN or AIN + AOM group (Figure 1C).

### 2.2. Diet and AOM Interaction 

**Figure 2:**
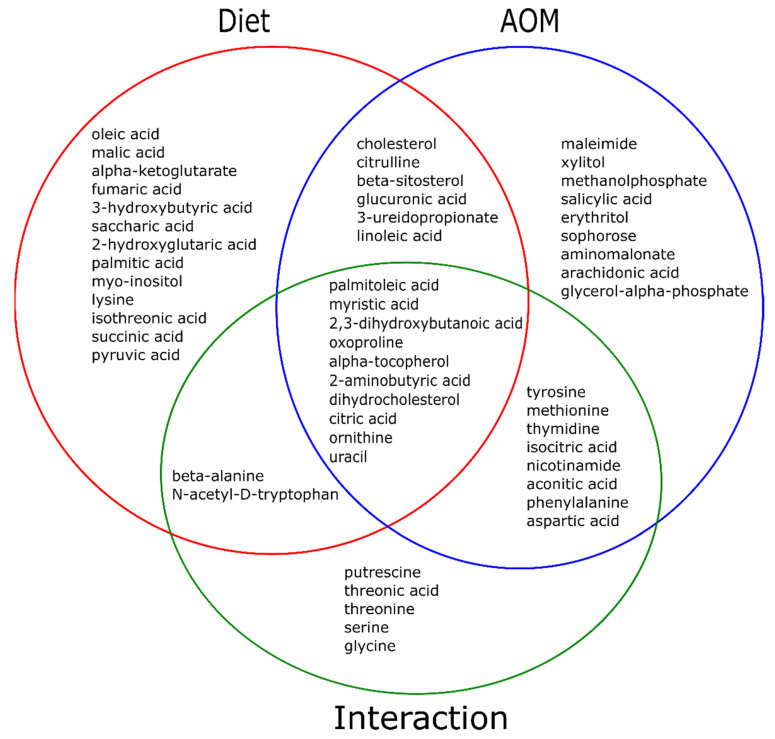
The interaction effect of diet and AOM on 53 plasma metabolites. Plot of significant interacting metabolites (green circle, 25 of 53 metabolites) by diet vs. AOM interaction, and two-way ANOVA between diet (red circle) and AOM (blue circle) with an FDR-adjusted *p* < 0.05. Mice without AOM treatment (control) *n* = 10/group (AIN or HFD group). Mice with AOM treatment, *n* = 15/group (AIN + AOM or HFD + AOM group).

To visualize the effect of diet and AOM interaction on the abundance of these 53 metabolites, a two-way ANOVA (including interaction tests) was performed. In Figure 2, there were 25 metabolites (green circle) that exhibited overall diet x AOM interactions in which (1) the concentration of 12 metabolites (the overlapped green and red circles) differed due to different diets while the concentration of 18 metabolites (the overlapped green and blue circles) differed because of AOM treatment; (2) the concentration of 10 metabolites (the overlapped green, red and blue circles) differed due to both diet and AOM treatment.

These 25 interactive metabolites included (1) the concentrations of alpha-tocopherol, dihydrocholesterol and nicotinamide were increased (> 58%) in the HFD group compared to the other 3 groups; in contrast, the concentrations of citric acid and myristic acid were decreased at least 23% in the HFD group compared to the other 3 groups (Table 1). These 5 metabolites comprise 50% of component 1 (Figure 1B); (2) the concentrations of oxoproline, 2-aminobutyric acid, uracil and ornithine were increased (> 29%) in the HFD + AOM group compared to the other 3 groups while palmitoleic acid was decreased (> 80%) in the (HFD or HFD + AOM) group compared to the (LFD or LFD + AOM) group (Table 1). These 5 metabolites consist 50% of component 2 (Figure 1C).

### 2.3. Metabolic Pathways of Altered Metabolites

**Figure 3:**
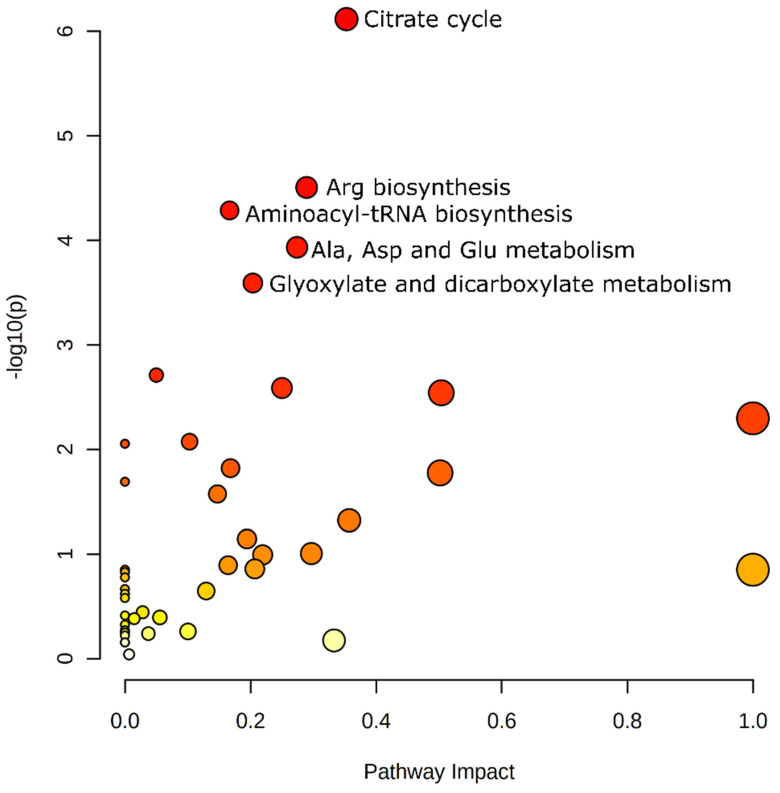
Pathway analysis of 53 differential metabolites and five major metabolic pathways *: (1) citrate cycle; (2) Arg biosynthesis; (3) aminoacyl-tRNA biosynthesis; (4) Ala, Asp, and Glu metabolism; (5) glyoxylate and dicarboxylate metabolism. * Only the pathways with statistical significance are highlighted.

To examine the biological significance of these altered 53 metabolites, we determined the potential pathways using the metabolite set enrichment analysis. These pathways (Figure 3, Table 2) included (1) the citric acid cycle: oxoglutaric acid, succinic acid, isocitric acid, *cis*-aconitic acid, citric acid, pyruvic acid, fumaric acid; (2) arginine biosynthesis: citrulline, aspartic acid, ornithine, oxoglutaric acid, fumaric acid; (3) aminoacyl-tRNA biosynthesis: phenylalanine, glycine, aspartic acid, serine, methionine, lysine, threonine, tyrosine; (4) alanine, aspartate, and glutamate metabolism: aspartic acid, citric acid, fumaric acid, pyruvic acid, succinic acid, oxoglutaric acid; and (5) glyoxylate and dicarboxylate metabolism: *cis*-aconitic acid, citric acid, serine, glycine, isocitric acid, pyruvic acid (Figure 3, Table 1 and Table 2).

### 2.4. Correlation between Colonic ACF and Dihydrocholesterol/Cholesterol

**Figure 4:**
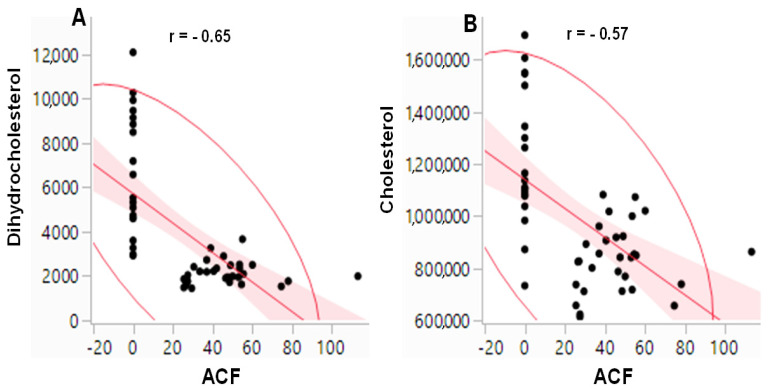
Scatterplot analysis of the correlation between (**A**) the number of ACF and the relative concentrations (quantifier ion peak heights) of dihydrocholesterol; (**B**) the number of ACF and the relative concentrations (quantifier ion peak heights) of cholesterol. With *n* = 50 each set, both (**A**,**B**) show an inverse association with a significant Spearman correlation (*p* < 0.0001), respectively.

A scatterplot analysis was used to determine the correlation between the number of ACF and the concentration of dihydrocholesterol/cholesterol. The AOM-induced colonic ACF data were taken from our previous study [7], and dihydrocholesterol/cholesterol data were from the current study (Table 1 and Table S1). The analysis (Figure 4) showed that there was an inverse association between the ACF number and dihydrocholesterol/cholesterol concentrations.

## 3. Discussion

Epidemiological and experimental data suggest that obesity increases colon cancer risk [2,3,6,7,8,9]. Our previous data demonstrate that a HFD promotes colonic ACF formation with increased levels of circulating proinflammatory cytokines in a mouse model [7]. In the present study, we found that 53 of 144 identified metabolites were different between the 4 experimental groups (Table 1, Figure S1). These data (1) confirm the hypothesis that AOM treatment causes different plasma metabolomic responses in AOM mouse colon cancer model fed with the HFD compared to the AIN; (2) support the notion that metabolomic fingerprints in the plasma may be used as potential markers for identifying changes in the connection between diet and colon cancer [13].

The separation between the HFD and HFD + AOM treatment groups but not the AIN and AIN + AOM groups (Figure 1A) indicates that AOM-induced colonic ACF formation altered the plasma metabolome to a greater extent in mice fed a HFD compared to the AIN. Compared to Component 2 of the sPLS-DA, Component 1 played a greater role (Figure 1B,C) in separating treatment groups [14,15]. The concentrations of alpha-tocopherol, nicotinamide, and beta-sitosterol were higher in the HFD group compared to the AIN group (Table 1, Figure 1B), which may be due to the increased dietary intakes (e.g., the alpha-tocopherol and beta-sitosterol in corn-oil) and homeostatic adaptation during the HFD consumption (e.g., nicotinamide) [7,16]. This observation is initially counterintuitive given the fact that alpha-tocopherol, beta-sitosterol, and nicotinamide are potential anticancer compounds [17,18,19]. For example, alpha-tocopherol exhibits superior antioxidant and anti-inflammatory properties via downregulation of transcription factor NF-kB activation [17]; beta-sitosterol and nicotinamide modulate multiple cell signaling pathways, including cell proliferation, angiogenesis, metastasis, inflammation, genomic stability, and immune-response [19,20,21]. However, compared to the AIN, the proinflammatory impact of HFD-induced obesity may outweigh the beneficial effects of these compounds (e.g., alpha-tocopherol); subsequently, AOM treatment induces a higher number of colonic ACF in the HFD group compared to the AIN group [7].

As colonic ACF formation is critically dependent on AOM bioactivation by the gut bacteria without affecting the daily food intake [7,22,23], the higher colonic ACF number may reduce the uptake of alpha-tocopherol, beta-sitosterol, and nicotinamide to a greater extent in the HFD + AOM group compared to the AIN + AOM group [7,24]. Thus, a low uptake efficacy of these beneficial compounds in the HFD + AOM group might further promote the vicious cycle of AOM-induced colonic ACF formation.

There are epidemiological reports on an inverse relationship between plasma cholesterol levels and certain colon cancer risk populations (e.g., during the 10 years preceding the cancer) [25,26,27]. In line with these epidemiological data [25,26,27], the concentrations of dihydrocholesterol and cholesterol in the AIN + AOM and HFD + AOM groups were decreased by 62% and 21% and 75% and 39% when compared to the AIN and HFD groups, respectively (Table 1, Figure 1B). The connection between declining serum cholesterol concentrations and colon cancer [25,26,27] may involve several factors. First, epithelial cell mutations occur during colonic tumorigenesis cause a decrease in intestinal cholesterol uptake [24,28]. Second, to meet the increasing demand for cell proliferation, cancer cells significantly enhance cholesterol absorption and synthesis [29,30]. Third, an intestinal cachexia may be a secondary result of metabolic and nutritional change in advanced tumorigenesis [27].

Dihydrocholesterol biosynthesis occurs through two major pathways: (1) unabsorbed cholesterol in the colon is biohydrogenated to dihydrocholesterol via bacterial enzymes [31,32], and (2) dihydrocholesterol is synthesized via a pathway with 7 alpha-hydroxylated C27-steroids as substrates in the liver [33]. Our data demonstrate that the levels of both plasma dihydrocholesterol (62 to 75% decrease) and cholesterol (21 to 39% decrease) are sensitive markers, which is inversely correlated to AOM-induced colonic aberrant crypt formation in the AIN and HFD groups (Figure 4) [7]. Because cholesterol is a precursor of dihydrocholesterol in the biosynthetic pathway [31,32,33], and the diet and AOM exhibit interactive effects on dihydrocholesterol but not cholesterol (Figure 2), these molecular events may account for a greater decrease of dihydrocholesterol concentrations compared to that of total plasma cholesterol (Table 1). As dihydrocholesterol is widely used as a surrogate marker of cholesterol synthesis and absorption [34], our data suggest that plasma dihydrocholesterol may be a more sensitive marker than cholesterol (62 to 75% vs. 21 to 39% decrease) for epidemiological studies [25,26,27] on colon cancer, although future human studies are needed to verify this finding.

Further functional analysis demonstrates that 53 plasma metabolites were involved in five major metabolic pathways (Figure 3, Table 2). First, the citrate cycle is a critical metabolic pathway that utilizes glucose, amino acids, and fatty acids. Emerging evidence demonstrates that cancer cells heavily rely on the citrate cycle for energy production and macromolecule synthesis [35]. Consequently, an altered citrate cycle pathway was detected in this study (Figure 3, Table 2), which is consistent with the serum metabolomic data from colon cancer patients [36,37]. Further studies are warranted to determine the connection between human colon cancer and plasma metabolomic profiles of the citrate cycle pathway in the context of obesity. Second, arginine biosynthesis and aminoacy*l-*tRNA biosynthesis are two pathways, which are involved in endothelial cell migration and angiogenesis in tumorigenesis [38,39]. However, there are scant data examining plasma metabolomic profiling in this regard. A change of these two pathways (Figure 3, Table 2) provides new insights into the plasma metabolome. Third, (a) alanine, aspartate, and glutamate metabolism and (b) glyoxylate and dicarboxylate metabolism are the other two pathways with no immediate functional connections with colon cancer. These two pathways are important for cellular homeostatic regulation because alanine, aspartate, and glutamate are derived from intermediates of central metabolism such as the citrate cycle pathway [40,41], a change in these two pathways (Figure 3, Table 2) may be the secondary results from the citrate cycle pathway.

In this report, although our data on differential plasma metabolomic responses are interesting, there are a few limitations. For example, our plasma metabolomic data show only a disease-associated relationship but not a causal relationship, and these data are from a single-time-point experiment. Because the cause of colon cancer is involved multiple metabolic pathways (collective effects), it is currently difficult for us to experimentally validate one or a few disease-causal metabolites. However, in the future, we will be able to shed some light on disease-causal metabolites if more metabolomic data/studies (with multiple experimental time points) are available.

## 4. Materials and Methods

### 4.1. Animals, Diets, and AOM Treatment

Three- to four-week-old male C57BL/6 mice (Harlan, Madison, WI, USA) were individually housed in Plexiglas^TM^ ventilated cages within a pathogen-free facility that maintained a 12 h light/dark cycle and a temperature of 22 ± 1 °C. Mice were given free access to food and deionized water. This study was approved by the Animal Care and Use Committee of the Grand Forks Human Nutrition Research Center (Protocol Code: HZ13M2, 2013), and animals were maintained in accordance with NIH guidelines for the care and use of laboratory animals. The study design, diet composition, and preparation have been previously reported [7]. Briefly, C57BL/6 mice were fed either an AIN or HFD (*n* = 25/group). On Week 3, within a given diet group, mice received either weekly intraperitoneal injection of the colon carcinogen, AOM (Sigma, St. Louis, MO, USA) (*n* = 15/group), at a concentration of 8 mg/kg body weight [42] or phosphate-buffered saline (PBS, pH = 7.4) carrier solution (*n* = 10/group) for 4 weeks. At the termination of the experiment, mice were fasted for 6 h and then euthanized with a mixture of ketamine and xylazine (100 mg/kg body weight). Plasma samples were collected at the end of the study (Week 14) and stored at −80 °C for metabolomic analysis.

### 4.2. Plasma Metabolomics

Metabolomic analysis was performed at the West Coast Metabolomics Center (University of California, Davis Genomic Center, Davis, CA, USA) [14,15]. Plasma samples were extracted and derivatized by silylation and methyloximation and analyzed by GC-TOF-MS for untargeted metabolomics. Data were processed at the West Coast Metabolomics Center using the BinBase database [43]. Metabolite quantifier ion peak heights were normalized to the sum intensities of all known compounds and used for the follow-up statistical analyses (Table S1).

### 4.3. Statistical and Bioinformatic Analysis

Bioinformatic analysis: to avoid a skewed distribution, obtained (peak intensity) data were normalized by Log10 transformation with an autoscaling method which is highly recommended for most peak intensity data [44,45]. In addition, the suitability (our data distribution) of the above-normalized method was the best when compared to other available normalized methods (e.g., Pareto scaling) using the MetaboAnalyst software (version 5.0, McGill University, Sainte Anne de Bellevue, Quebec, Canada) [44,45]. The metabolite group separation and functional pathways were analyzed by sPLS-DA, heatmap clustering, and metabolite set enrichment analysis (MSEA) using the MetaboAnalyst software [44,45], respectively. The effects of diet (AIN or HFD) and AOM treatment (with or without) on the relative abundance of plasma metabolites were analyzed using a two-way analysis of variance (ANOVA) corrected by a false discovery rate (FDR) of 0.05 and Tukey’s contrasts for post hoc comparisons. Results are given as mean ± standard deviation (SD). JMP V14 (SAS Institute Inc., Cary, NC, USA) and MetaboAnalyst software (version 4.0, McGill University, Sainte Anne de Bellevue, QC, Canada) were used for all statistical analyses [44,45].

## 5. Conclusions

Our data demonstrated that AOM, a commonly used carcinogen in a chemical-induced colon cancer model, altered the plasma metabolome to a greater extent in mice fed the HFD compared to the AIN. These plasma metabolites were involved in five major metabolic pathways included (1) citrate cycle, (2) Arg biosynthesis, (3) aminoacyl-tRNA biosynthesis, (4) Ala, Asp, and Glu metabolism, and (5) glyoxylate and dicarboxylate metabolism. Our newly identified (inverse) association between plasma dihydrocholesterol levels and AOM-induced colonic ACF formation in a mouse model warrants future exploration as one of the unique plasma “multiple analytes” at the quest of human colon cancer biomarker search.

## Figures and Tables

**Table 1:** The effect of HFD feeding and AOM treatment on the abundance of plasma metabolites.

Metabolites	AIN	AIN + AOM	HFD	HFD + AOM
Palmitoleic acid	1.00 ± 0.40 ^a^	1.11 ± 0.27 ^a^	0.19 ± 0.05 ^b^	0.30 ± 0.11 ^b^
Myristic acid	1.00 ± 0.18 ^a^	1.02 ± 0.10 ^a^	0.57 ± 0.06 ^c^	0.74 ± 0.12 ^b^
Oleic acid	1.00 ± 1.46 ^bc^	0.73 ± 1.32 ^c^	1.99 ± 0.42 ^ab^	2.71 ± 0.95 ^a^
Malic acid	1.00 ± 0.26 ^a^	1.23 ± 0.42 ^a^	0.63 ± 0.08 ^b^	0.60 ± 0.12 ^b^
Beta-sitosterol	1.00 ± 0.35 ^b^	0.46 ± 0.11 ^c^	2.22 ± 0.65 ^a^	0.64 ± 0.10 ^bc^
Oxoproline	1.00 ± 0.14 ^b^	1.08 ± 0.15 ^b^	1.13 ± 0.17 ^b^	1.42 ± 0.21 ^a^
Alpha-tocopherol	1.00 ± 0.23 ^b^	0.67 ± 0.10 ^c^	1.94 ± 0.46 ^a^	0.84 ± 0.17 ^bc^
Alpha-ketoglutarate	1.00 ± 0.22 ^a^	1.15 ± 0.34 ^a^	0.72 ± 0.09 ^b^	0.63 ± 0.13 ^b^
Fumaric acid	1.00 ± 0.14 ^a^	1.17 ± 0.32 ^a^	0.76 ± 0.05 ^b^	0.75 ± 0.11 ^b^
Citric acid	1.00 ± 0.24 ^ab^	1.08 ± 0.14 ^a^	0.68 ± 0.07 ^c^	0.88 ± 0.10 ^b^
3-hydroxybutyric acid	1.00 ± 0.64 ^c^	1.32 ± 0.62 ^bc^	2.08 ± 0.50 ^a^	1.80 ± 0.57 ^ab^
Saccharic acid	1.00 ± 0.18 ^c^	1.14 ± 0.21 ^bc^	1.49 ± 0.36 ^a^	1.33 ± 0.21 ^ab^
Citrulline	1.00 ± 0.21 ^a^	0.87 ± 0.17 ^ab^	0.77 ± 0.13 ^bc^	0.64 ± 0.12 ^c^
2-hydroxyglutaric acid	1.00 ± 0.13 ^ab^	1.12 ± 0.17 ^a^	0.86 ± 0.11 ^b^	0.87 ± 0.14 ^b^
2-aminobutyric acid	1.00 ± 0.37 ^b^	0.87 ± 0.36 ^b^	0.88 ± 0.24 ^b^	1.58 ± 0.54 ^a^
Palmitic acid	1.00 ± 0.15 ^ab^	1.09 ± 0.14 ^a^	0.86 ± 0.08 ^b^	0.88 ± 0.13 ^b^
3-ureidopropionate	1.00 ± 0.29 ^b^	1.35 ± 0.49 ^b^	1.19 ± 0.47 ^b^	2.02 ± 0.55 ^a^
Myo-inositol	1.00 ± 0.24 ^b^	0.97 ± 0.13 ^b^	1.37 ± 0.32 ^a^	1.09 ± 0.27 ^b^
Dihydrocholesterol	1.00 ± 0.26 ^b^	0.48 ± 0.12 ^c^	1.89 ± 0.55 ^a^	0.48 ± 0.12 ^c^
Glucuronic acid	1.00 ± 0.33 ^c^	1.98 ± 0.51 ^ab^	1.32 ± 0.37 ^bc^	2.48 ± 0.96 ^a^
Lysine	1.00 ± 0.37 ^a^	0.77 ± 0.22 ^ab^	0.57 ± 0.25 ^b^	0.58 ± 0.15 ^b^
Cholesterol	1.00 ± 0.14 ^b^	0.79 ± 0.14 ^c^	1.32 ± 0.22 ^a^	0.81 ± 0.12 ^c^
Isothreonic acid	1.00 ± 0.09 ^b^	1.07 ± 0.08 ^b^	1.22 ± 0.20 ^a^	1.03 ± 0.06 ^b^
Uracil	1.00 ± 0.33 ^b^	1.04 ± 0.15 ^b^	0.95 ± 0.17 ^b^	1.29 ± 0.23 ^a^
Ornithine	1.00 ± 0.27 ^b^	1.39 ± 0.62 ^b^	1.05 ± 0.46 ^b^	2.04 ± 0.33 ^a^
Succinic acid	1.00 ± 0.21 ^ab^	1.27 ± 0.47 ^a^	0.87 ± 0.11 ^b^	0.87 ± 0.24 ^b^
N-acetyl-d-tryptophan	1.00 ± 0.22 ^a^	1.00 ± 0.26 ^a^	0.68 ± 0.22 ^b^	0.87 ± 0.18 ^ab^
Pyruvic acid	1.00 ± 0.45 ^ab^	1.15 ± 0.65 ^a^	0.58 ± 0.18 ^b^	0.67 ± 0.34 ^b^
2,3-dihydroxybutanoic acid	1.00 ± 0.19 ^b^	1.11 ± 0.12 ^ab^	1.00 ± 0.12 ^b^	1.29 ± 0.24 ^a^
Linoleic acid	1.00 ± 0.26 ^b^	1.10 ± 0.26 ^ab^	0.99 ± 0.20 ^b^	1.41 ± 0.50 ^a^
Beta-alanine	1.00 ± 0.32 ^a^	0.97 ± 0.33 ^a^	0.60 ± 0.21 ^b^	0.86 ± 0.23 ^ab^
Maleimide	1.00 ± 0.13 ^b^	0.88 ± 0.14 ^b^	1.26 ± 0.14 ^a^	0.85 ± 0.14 ^b^
Isocitric acid	1.00 ± 0.17 ^a^	1.15 ± 0.23 ^a^	0.77 ± 0.11 ^b^	1.08 ± 0.14 ^a^
Tyrosine	1.00 ± 0.19 ^a^	1.12 ± 0.20 ^a^	0.75 ± 0.22 ^b^	1.06 ± 0.14 ^a^
Xylitol	1.00 ± 0.54 ^b^	1.58 ± 0.42 ^a^	0.90 ± 0.23 ^b^	2.03 ± 0.65 ^a^
Nicotinamide	1.00 ± 0.26 ^b^	0.92 ± 0.20 ^bc^	1.58 ± 0.24 ^a^	0.76 ± 0.15 ^c^
Putrescine	1.00 ± 0.34 ^a^	0.77 ± 0.25 ^ab^	0.70 ± 0.10 ^b^	0.70 ± 0.12 ^b^
Threonic acid	1.00 ± 0.15 ^b^	1.16 ± 0.24 ^ab^	1.39 ± 0.22 ^a^	1.01 ± 0.24 ^b^
Methanolphosphate	1.00 ± 0.12 ^b^	0.69 ± 0.11 ^c^	1.18 ± 0.15 ^a^	0.65 ± 0.09 ^c^
Threonine	1.00 ± 0.23 ^a^	0.76 ± 0.13 ^b^	0.74 ± 0.16 ^b^	0.75 ± 0.12 ^b^
Serine	1.00 ± 0.22 ^ab^	0.82 ± 0.14 ^b^	0.80 ± 0.21 ^b^	1.02 ± 0.14 ^a^
Glycine	1.00 ± 0.32 ^a^	0.82 ± 0.12 ^ab^	0.78 ± 0.09 ^b^	0.80 ± 0.12 ^b^
Salicylic acid	1.00 ± 0.16 ^ab^	1.15 ± 0.21 ^ab^	0.95 ± 0.16 ^b^	1.20 ± 0.19 ^a^
Erythritol	1.00 ± 0.13 ^b^	1.13 ± 0.07 ^a^	1.03 ± 0.10 ^b^	1.08 ± 0.06 ^ab^
Sophorose	1.00 ± 0.32 ^ab^	0.88 ± 0.29 ^b^	1.28 ± 0.44 ^a^	0.78 ± 0.18 ^b^
Thymidine	1.00 ± 0.21 ^ab^	1.03 ± 0.24 ^a^	0.79 ± 0.13 ^b^	1.20 ± 0.20 ^a^
Aminomalonate	1.00 ± 0.34 ^a^	0.76 ± 0.14 ^bc^	0.93 ± 0.25 ^ab^	0.67 ± 0.11 ^c^
Arachidonic acid	1.00 ± 0.11 ^a^	0.71 ± 0.14 ^b^	1.06 ± 0.16 ^a^	0.60 ± 0.10 ^b^
Aconitic acid	1.00 ± 0.35 ^ab^	1.09 ± 0.32 ^a^	0.75 ± 0.30 ^b^	1.14 ± 0.25 ^a^
Phenylalanine	1.00 ± 0.19 ^ab^	0.97 ± 0.24 ^ab^	0.81 ± 0.31 ^b^	1.13 ± 0.20 ^ab^
Aspartic acid	1.00 ± 0.33 ^ab^	1.01 ± 0.28 ^ab^	0.72 ± 0.20 ^b^	1.13 ± 0.27 ^a^
Methionine	1.00 ± 0.24 ^ab^	0.94 ± 0.26 ^ab^	0.73 ± 0.30 ^b^	1.08 ± 0.23 ^a^
Glycerol-alpha-phosphate	1.00 ± 0.29 ^a^	0.79 ± 0.08 ^b^	0.89 ± 0.14 ^ab^	0.79 ± 0.10 ^b^

Values are means ± SDs, *n* = 10/group (AIN or HFD group) and *n* = 15/group (AIN + AOM or HFD + AOM group). Data from the HFD, AIN + AOM, and HFD + AOM groups were converted to fold changes compared to the AIN group. For a given metabolite, if two values share at least one letter, then the difference between them is not statistically significant. However, if they do not have a letter in common, then the difference between them is statistically significant, *p* < 0.05 adjusted by the FDR method.

**Table 2:** The major metabolic pathways * involved in 53 differential metabolites (between experimental groups).

KEGG Pathway	Number of Metabolites Identified	*p* *	Impact **
Citrate cycle (TCA cycle)	7	<0.0001	0.35
Arg biosynthesis	5	<0.003	0.29
Aminoacyl-tRNA biosynthesis	8	<0.005	0.17
Ala, Asp, and Glu metabolism	6	<0.01	0.27
Glyoxylate and dicarboxylate metabolism	6	<0.03	0.20

* Only the pathways with statistical significance are listed, and *p*-values are obtained by the over-representation analysis and adjusted by Holm and FDR methods. ** Impact is the pathway impact score obtained by the pathway topology analysis.

## Data Availability

All plasma metabolomics data used in this work are available as a Supplementary excel file (Table S1).

## Supplementary Materials

The following are available online at https://www.mdpi.com/article/10.3390/metabo11070448/s1. Figure S1: Hierarchical clustering heatmap analysis of 53 differential metabolites between experimental groups. Each column represents the metabolite abundance of one animal plasma sample. Without AOM treatment (control), *n* = 10/group (AIN or HFD group). With AOM, treatment *n* = 15/group (AIN + AOM or HFD + AOM group). Table S1: Metabolites identified in plasma from C57BL/6 mice fed the AIN93G (AIN) or the high-fat diet (HFD) and with or without AOM treatment.

## Abbreviations

AC, aberrant crypt; ACF, aberrant crypt foci; AIN, a modified AIN93G formulation; Ala, alanine; ANOVA, analysis of variance; AOM, azoxymethane; Arg, arginine; Asp, aspartate; FDR, false discovery rate; Glutamate, Glu; sPLS-DA, sparse partial least-squares discriminant analysis; 2D, two-dimensional; GC-TOFMS; time-of-flight mass spectrometry; KEGG, Kyoto encyclopedia of genes and genomes; TCA cycle, citrate cycle.
