# Supplementary figures and images for: Azoxymethane Alters the Plasma Metabolome to a Greater Extent in Mice Fed a High-Fat Diet Compared to an AIN-93 Diet
Source: Metabolites. 2021 Jul 9;11(7):448. doi: 10.3390/metabo11070448 (PMC8307161; doi:10.3390/metabo11070448)

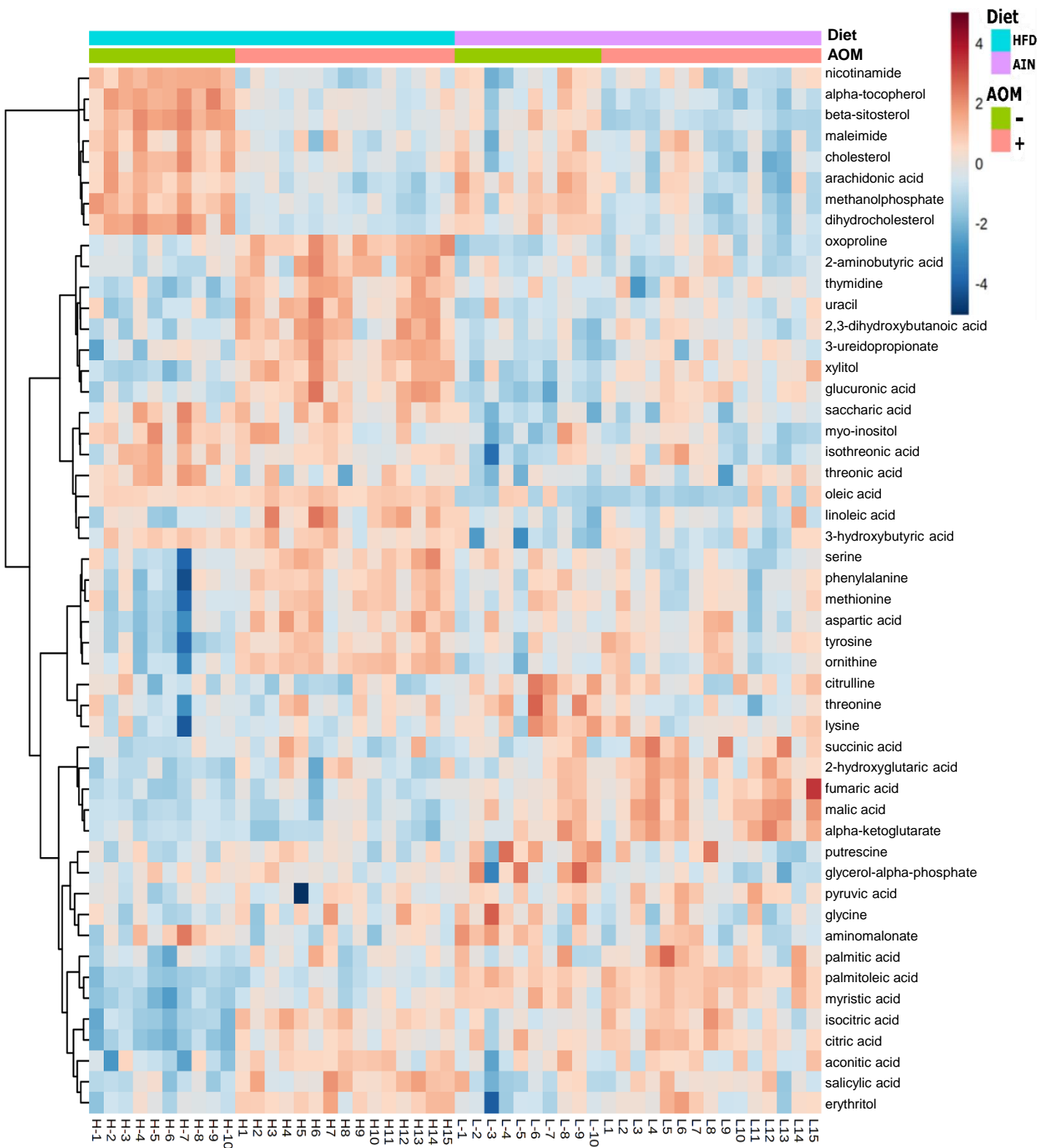

Supplementary Figure S1

Supplement: Supplementary file 1 [file metabolites-11-00448-s001.zip › Supplementary Figure S1.pdf]
